# Racial and ethnic differences in physical activity among mothers of young children: 2011–2018 NHANES

**DOI:** 10.1186/s12905-023-02591-x

**Published:** 2023-08-18

**Authors:** Soyang Kwon, Milkie Vu, Nina M. Wetoska, Tami R. Bartell

**Affiliations:** 1 Department of Pediatrics, Northwestern University, 225 E Chicago Ave. Box 157, Chicago, IL 60611 USA; 2 Department of Preventive Medicine, Northwestern University, 680 N. Lakeshore Dr. Suite 1400, Chicago, IL 60611 USA; 3 Buehler Center for Health Policy and Economics, Northwestern University, Chicago, USA; 4 Patrick M. Magoon Institute for Healthy Communities, Ann & Robert H Lurie Children’s Hospital of Chicago, 225 E Chicago Ave. Box 157, Chicago, IL 60611 USA

**Keywords:** Motherhood, Women, Exercise, Race, Ethnicity, Children

## Abstract

**Background:**

American women tend to reduce physical activity (PA) during the transition to motherhood. Their main barrier to participation in PA is lack of time due to new/increased parenting and housework responsibilities. Because there are known racial/ethnic variations in time spent on housework among American women, their PA changes during the transition to motherhood might also differ by racial/ ethnic background. This study aimed to compare PA between American mothers of young child(ren) under age 5 years (YC) and American women without children by their racial/ethnic background.

**Methods:**

Secondary data analyses were conducted using 2011–2018 US National Health and Nutrition Survey data. The study sample included 4,892 women aged 20–45 years (Asian n = 760; Black n = 1,162; Hispanic n = 1,324; White n = 1,646). Participants completed a Physical Activity Questionnaire that asked about participation in transportation and leisure-time moderate- and vigorous-intensity PA (MVPA; minutes/week). Multivariable regression analyses were conducted to compare MVPA among women living without children and with YC (no older children) in each of the racial/ethnic groups.

**Results:**

Overall, the prevalence of physical inactivity, defined as zero minutes of MVPA in a typical week, was 43% (95% CI = 38–49%) vs. 32% (95% CI = 29–35%) among women living with YC vs. without children. The adjusted odds of physical inactivity for women living with YC, compared to women living without children, was significantly higher among Asian (OR = 2.08 [95% CI = 1.37–3.17]) and White women (OR = 1.63 [95% CI = 1.11–2.38]), while it was statistically insignificant among Hispanic and Black women. Among women who reported participating in MVPA, Asian women living with YC had 35 fewer minutes/week of MVPA than their counterparts living without children (p = 0.06), while other racial and ethnic groups showed no significant differences.

**Conclusions:**

American mothers of YC were less likely to engage in transportation or leisure-time MVPA, compared to those living without children. This association was particularly strong among Asian women. The study results suggest that a PA reduction in the transition to motherhood may be particularly large among Asian American women, calling for targeted efforts for PA promotion among Asian American mothers of YC; e.g., culturally-tailored community-based physical activity programs for Asian American mothers.

**Supplementary Information:**

The online version contains supplementary material available at 10.1186/s12905-023-02591-x.

## Background

Physical activity (PA) provides numerous physical and mental health benefits, including but not limited to obesity prevention [1, 2]. Although American women perform better than American men in most Leading Health Indicators, PA and obesity reduction are two Leading Health Indicators for which women lag behind men [3]. The gender difference in PA has been observed across socioeconomic classes [4, 5]. PA promotion among women is, therefore, a key health promotion strategy that could have a substantial impact on overall population health [6].

The life transition to parenthood has been reported to be associated with a PA reduction among American adults, and with a greater reduction among women than men [7, 8]. Several studies [9–12] have reported that a main barrier to PA among mothers of young children (YC; under age 5 years) is a lack of time due to new and/or increased parenting responsibilities. A greater PA reduction in the transition to parenthood among women than men could be partly explained by a greater increase in women’s time spent on housework as compared to men [13]. Furthermore, significant variations in women’s and men’s housework time were found across racial and ethnic groups in United States (US) populations, revealing that women’s contribution to housework (relative to men’s) was larger among Asian and Hispanic groups, compared to Black or White populations [13]. Understanding changes in PA behavior during this critical life transition across racial and ethnic groups will help to identify a subgroup of women at risk of PA reduction and design a targeted PA promotion intervention.

We hypothesized that PA reduction during the transition to motherhood would be larger among Asian and Hispanic American women, compared to Black and White American women. These four racial and ethnic groups were selected consistent with a prior study [13]. To test the hypothesis, this study aimed to examine PA among American mothers of young children under age 5 years [YC], in comparison with American women without children by their racial and ethnic background.

## Methods

### Study sample

A secondary data analysis was conducted using data from the 2011–2012, 2013–2014, 2015–2016, and 2017–2018 US National Health and Nutrition Examination Survey (2011-18 NHANES). NHANES is a national survey that includes a representative sample of the US population, using a complex staged sampling method. NHANES is comprised of two components: an interview and an examination. The NHANES in-person interview was conducted in the participant’s home. The NHANES examination was conducted in the Mobile Examination Center (MEC). The 2011-18 NHANES cycles were approved by the National Center for Health Statistics Ethics Review Board (protocol #2011-17 and protocol #2018-01).

For this secondary data analysis we identified 5,111 woman participants aged 20–45 years from the 2011-18 NHANES datasets. This age range was selected to include most adult mothers of YC, considering the age range for reproductive years among American women giving birth [14]. Among the 5,111 women, we excluded 219 (3.9%) who self-identified as multi or other races than Asian, Black, Hispanic, or White, resulting in 4,892 women for data analysis. We did not exclude women who reported being pregnant (n = 247), because PA is recommended even during pregnancy; at least 150 min of moderate-intensity PA per week [2].

### Measurements

Exposure: Type of children living in the same household. During the interview, a demographic survey was administered, which included questions about the total number of YC living in the same household and the total number of older children aged 6–17 years (OC) living in the same household with the participant. However, it did not ask about the relationships (e.g., child, relative) between the participant and the other household member(s). Therefore, adopting the approach used by Adamo et al. [15], we defined women who reported at least one YC and no OC living in the same household (women with YC, hereafter) as mothers of YC. In the same manner, we defined women who reported at least one OC and no YC living in the same household (women with OC, hereafter) as mothers of OC. Women who reported both YC and OC living in the same household (women with YC and OC, hereafter) were defined as mothers of YC and OC. Women who reported no children aged 17 years or under living in the same household (women without children, hereafter) served as a comparison group. As a result, the variable for the type of children living in the same household had four categories: no-children, YC, OC, and YC and OC (YC + OC).

Moderator: Racial and ethnic background. The demographic survey asked participants to self-identify their Hispanic origin (Hispanic or non-Hispanic) and race. Those who self-identified as Hispanic origin were classified as Hispanic, regardless of race. Those who self-identified as non-Hispanic origin were classified as Asian, Black, White, and multi or other race, based on their race response. The multi or other race group was excluded from this study.

Outcome: Physical activity. Among PA domains, this study focused on transportation and leisure-time PA, but not occupational PA or housework PA. Occupational PA was excluded to be aligned with the US Center for Disease Control and Prevention (CDC)’s report that evaluated the national prevalence of physical inactivity *outside of work* [16]. Also, in consideration of future intervention development, occupational PA is less modifiable as compared to transportation and leisure-time PA. Housework PA was not considered because it was not assessed in the 2011-18 NHANES PA questionnaire (PAQ).

During the interview, a PAQ was administered. The PAQ asked about the frequencies and durations of occupational, transportation, and leisure-time PA in a typical week. For transportation PA, participants were asked “In a typical week, on how many days do you walk or bicycle for at least 10 minutes continuously to get to and from places?”; and “How much time do you spend walking or bicycling for travel on a typical day?” For leisure-time PA, questions about engagement in vigorous-intensity and moderate-intensity PA were asked separately: “In a typical week, on how many days do you do vigorous-intensity sports, fitness, or recreational activities?; “How much time do you spend doing vigorous-intensity sports, fitness, or recreational activities on a typical day?”; “In a typical week, on how many days do you do moderate-intensity sports, fitness, or recreational activities that cause a small increase in breathing or heart rate such as brisk walking, bicycling, swimming, or volleyball for at least 10 minutes continuously?”; and “How much time do you spend doing moderate-intensity sports, fitness, or recreational activities on a typical day?”

Weekly transportation moderate- and vigorous-intensity PA (MVPA) minutes (minutes/week) were calculated by multiplying the frequency (days/week) and duration (minutes/day) of transportation PA. Weekly leisure-time MVPA minutes (minutes/week) were calculated by summing weekly leisure-time vigorous-intensity PA minutes and weekly leisure-time moderate-intensity PA minutes. Weekly transportation MVPA minutes and weekly leisure-time MVPA minutes were summed to calculate overall weekly MVPA minutes. Physical inactivity was defined as participating in zero minutes of overall MVPA in a typical week.

Confounding factors. We considered the following potential confounding factors: age in years, marital status, education, employment, pregnancy status, family income, and body mass index (BMI). During the interview, the demographic survey asked about age in years, marital status (categorized into married, living with a partner, and widowed/divorced/separated/never married), education level (categorized into ≤ high school graduate, some college or Associate in Arts degree, and ≥ college graduate), employment (categorized into employed and non-employed), pregnancy status (categorized into pregnant, not pregnant, and unknown), family income, and family size. A poverty index ratio, a ratio of family income to poverty, was calculated based on family size and income and then categorized into < 1.0 (below the current US poverty level; low income), 1 to < 3.0 (middle income), and ≥ 3.0 (high income). Anthropometry (height and weight) assessments were conducted in the MEC. BMI was calculated as weight (kg) divided by height squared (m^2^) and categorized into < 25, 25 to < 30, and ≥ 30 kg/m^2^.

### Statistical analysis

All analyses were conducted using SAS 9.4 survey procedure (Cary, NC), accounting for the complex sample design, such as weighting and clustering. Descriptive analyses, including distribution analyses, for all study variables were conducted. No missing data were found for any study variables, except family income and BMI categories which were treated using a median imputation method: those with missing data for the poverty index ratio (n = 444; 10%) were assigned to the middle-income category, and those with missing data for BMI (n = 236; 5%) were assigned to the middle category, 25 to < 30 kg/m^2^. The means and proportions of the PA variables were compared between women living with YC and without children in each of the four racial and ethnic groups, using 95% confidence intervals (CIs).

Multivariable logistic regression analyses were conducted, stratified by racial and ethnic groups to compare the probability of physical inactivity by the type of children living in the same household (reference: no-children group) within each racial and ethnic group. In the regression analyses, we included the following confounding factors as predictors: age in years, marital status, education level, employment, poverty index ratio category, and BMI category. Odds ratios (ORs) and 95% CIs were obtained from the logistic regression models. In sensitivity analysis, multivariable logistic regression analysis was repeated following the steps previously outlined but excluding participants who were pregnant. We also repeated the analysis excluding those with missing data for poverty index ratio or BMI to evaluate the impacts of the imputations on the regression analysis results. To explore a dose-response relationship between the number of children living in the household and physical inactivity, an exploratory analysis was conducted using the number of YC (0, 1, or ≥ 2) and the number of OC (0, 1, or ≥ 2) in the household as predictors in multivariable logistic regression models. To explore the effects of immigration status, logistic regression analysis was conducted, additionally including the immigration status variable, identified by birthplace (foreign-born vs. US-born).

Among those who reported engaging in at least one minute of MVPA in a typical week, multivariable linear regression analyses were conducted stratified by racial and ethnic group to compare weekly MVPA minutes by the type of children living in the same household (reference group: no-children). Because the distribution of the weekly MVPA minute variable showed a long tail on the right side due to outliers, values above the 99th percentile (> 720 min) were recoded as 720 min to improve the distribution. These linear regression analyses included the same set of predictors as the multivariable logistic regression analyses described above. A significance level was set at 0.05 (two-sided).

## Results

Among the 4,892 participants, 760 self-identified as Asian, 1,162 as Black, 1,324 as Hispanic, and 1,646 as White. Overall, 1,473 participants reported living without children in the same household, 874 with YC, 1,480 with OC, and 1,065 with YC and OC. The average age of the participants was 30, 30, 37, and 33 years for the no-children, YC, OC, and YC + OC groups, respectively. The characteristics of the study participants are presented in Table 1.

**Table 1:** Characteristics of woman participants aged 20–45 years in the 2011-18 US NHANES.

	No children (n = 1,473)	Younger child(ren) only (n = 874)	Older child(ren) only (n = 1,480)	Younger and older children (n = 1,065)
	n (%)	n (%)	n (%)	n (%)
**Race and ethnicity**				
Asian	284 (7.6)	149 (7.1)	195 (6.3)	132 (6.7)
Black	331 (12.2)	185 (13.2)	379 (16.4)	267 (17.0)
Hispanic	277 (13.8)	209 (18.7)	453 (23.1)	385 (31.3)
White	581 (66.4)	331 (61.0)	453 (54.2)	281 (45.0)
**Marital status**				
Married	393 (28.3)	480 (60.7)	752 (55.1)	559 (58.0)
Never married, widowed, divorced, or separated	871 (56.5)	251 (24.4)	583 (35.4)	325 (26.2)
Living with a partner	209 (15.2)	143 (14.9)	145 (9.5)	181 (15.8)
**Education**				
≤High school graduate/GED	327 (19.9)	310 (32.7)	584 (35.0)	495 (41.8)
Some college or Associate in Arts degree	531 (35.8)	309 (33.8)	559 (37.6)	367 (36.3)
≥College graduate	615 (44.3)	255 (33.5)	337 (27.1)	203 (21.9)
**Employment**				
Employed	1,105 (79.3)	524 (62.5)	1038 (71.5)	551 (54.2)
Unemployed	368 (20.7)	350 (37.5)	442 (28.5)	514 (45.8)
**Family income**				
Low	254 (14.0)	220 (20.1)	313 (15.8)	372 (29.8)
Middle	601 (36.8)	412 (45.8)	752 (45.9)	523 (48.8)
High	618 (49.2)	242 (34.1)	415 (38.3)	170 (21.4)
**Currently Pregnant**				
Yes	1,364 (92.9)	774 (88.1)	1,339 (89.3)	989 (93.6)
No	49 (3.3)	90 (11.1)	35 (1.9)	63 (5.5)
Unknown	60 (3.8)	10 (0.8)	106 (8.8)	13 (0.9)
**Body mass index (BMI)**				
< 25 kg/m^2^	607 (41.7)	318 (35.1)	413 (29.9)	293 (28.5)
25 ≤ to < 30 kg/m^2^	397 (28.2)	261 (29.3)	424 (29.1)	350 (32.8)
≥ 30 kg/m^2^	469 (30.1)	295 (35.6)	643 (41.0)	422 (38.7)

Unweighted sample size (n) and weighted percentage (%) are presented. GED, general education development test

**Fig. 1:**
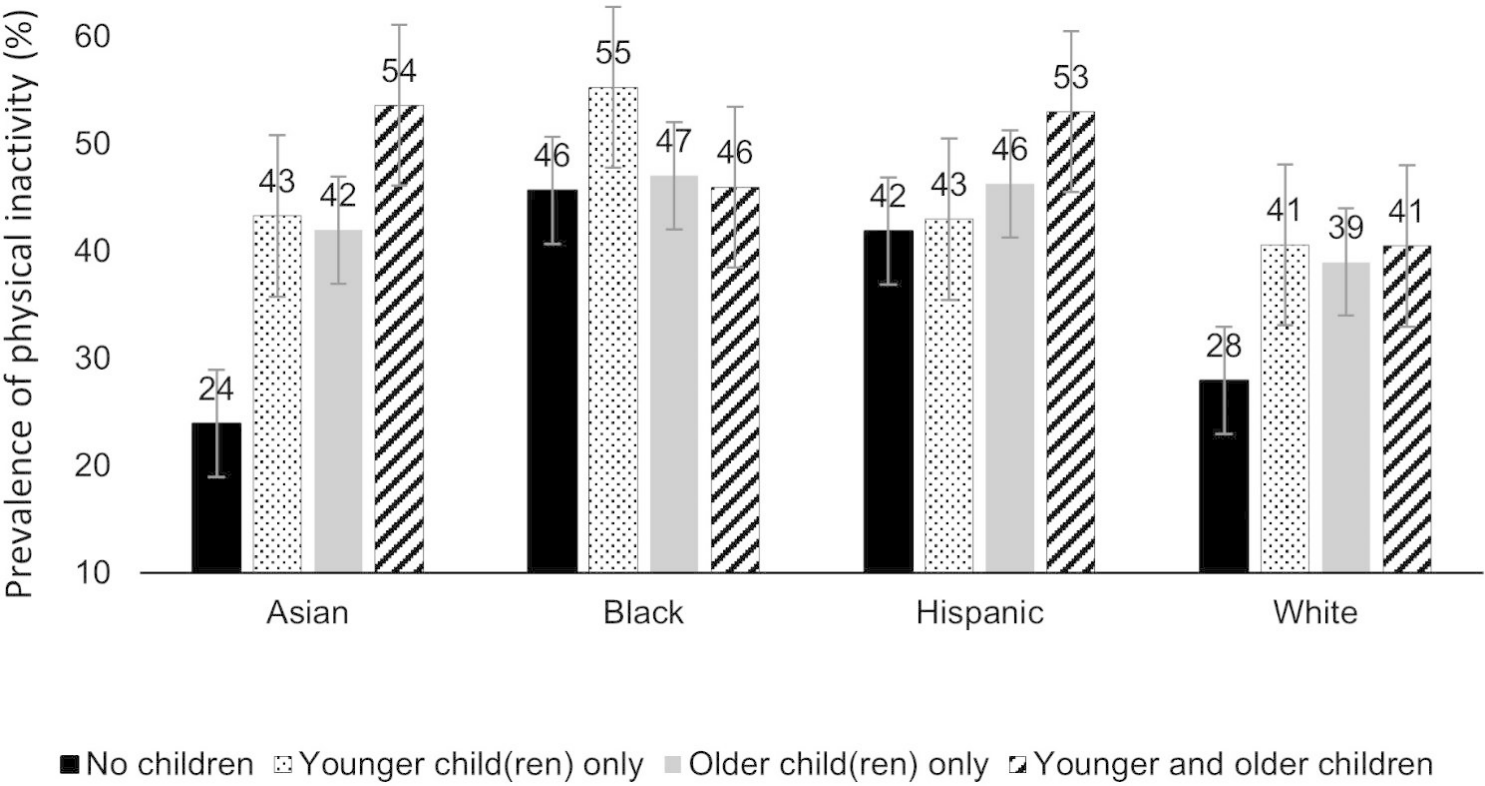
Prevalence and 95% confidence intervals of physical inactivity outside of work among woman participants aged 20–45 years in the 2011-18 US NHANES

Overall, the prevalence of physical inactivity was 32% (95% CI = 29, 35), 43% (95% CI = 38, 49), 42% (95% CI = 39, 45), and 46% (95% CI = 43, 50) in the no-children, YC, OC, and YC + OC groups, respectively. In the no-children group, Asian and White women (24% and 28%, respectively) had a significantly lower prevalence of physical inactivity, compared to Black and Hispanic women (46% and 42%, respectively; p < 0.05; Fig. 1). The prevalence of physical inactivity was significantly higher in the YC group, compared to the no-children group, among Asian, Black, and White women, but not among Hispanic women. Similarly, overall average weekly MVPA minutes were significantly lower in the YC group compared to the no-children group, among Asian, Black, and White women, but not among Hispanic women (Table 2).

**Table 2:** Levels of transportation and leisure-time physical activity among women participants aged 20–45 years in the 2011-18 US NHANES.

	No children (n = 1,473)	Younger child(ren) only (n = 874)	Older child(ren) only (n = 1,480)	Younger and older children (n = 1,065)
	Mean (95% CI)	Mean (95% CI)	Mean (95% CI)	Mean (95% CI)
**Transportation PA, minutes/week**				
Asian	48 (35, 60)	34 (21, 46)	35 (22, 48)	27 (17, 37)
Black	33 (20, 47)	31 (17, 44)	25 (17, 31)	27 (17, 36)
Hispanic	33 (23, 44)	42 (29, 54)	36 (29, 43)	32 (24, 40)
White	45 (35, 56)	22 (14, 31)	19 (12, 27)	35 (21, 49)
**Leisure-time MVPA, minutes/week**				
Asian	117 (103, 130)	65 (49, 80)	71 (58, 85)	50 (33, 68)
Black	70 (58, 83)	54 (40, 69)	71 (61, 81)	73 (56, 90)
Hispanic	102 (83, 122)	79 (61, 98)	87 (73, 101)	63 (55, 71)
White	133 (117, 148)	106 (88, 124)	100 (88, 111)	94 (74, 114)
**Sum of transportation and leisure-time MVPA, minutes/week**				
Asian	164 (147, 182)	98 (78, 118)	106 (86, 126)	78 (58, 98)
Black	102 (84, 119)	85 (68, 102)	95 (84, 107)	100 (81, 118)
Hispanic	135 (113, 156)	120 (97, 143)	122 (107, 138)	94 (81, 108)
White	176 (157, 196)	128 (108, 148)	119 (106, 132)	128 (104, 152)

CI, confidence interval; MVPA, moderate- and vigorous-intensity physical activity; PA, physical activity

After adjusting for confounding factors, the OR of physical inactivity was 2.08 (95% CI = 1.37, 3.17) for Asian women with YC and 1.63 (95% CI = 1.11, 2.38) for White women with YC, compared to their counterparts without children (Table 3). No significant association was found between the type of children living in the same household and physical inactivity among Black and Hispanic women, respectively. In a sensitivity analysis excluding women who reported a current pregnancy, the ORs of physical inactivity hardly changed: OR = 2.10 (95% CI = 1.31, 3.37) for Asian women, OR = 1.10 (95% CI = 0.72, 1.68) for Black women, OR = 1.04 (95% CI = 0.65, 1.66) for Hispanic women, and OR = 1.60 (95% CI = 1.09, 2.36) for White women. Similarly, in a sensitivity analysis excluding women who had missing data for family poverty index or BMI, the ORs of physical inactivity showed little change: OR = 1.96 (95% CI = 1.21, 3.17) for Asian women, OR = 1.21 (95% CI = 0.85, 1.72) for Black women, OR = 1.03 (95% CI = 0.63, 1.69) for Hispanic women, and OR = 1.60 (95% CI = 1.06, 2.39) for White women.

**Table 3:** Adjusted odds ratios of physical inactivity outside of work among woman participants aged 20–45 years in the 2011-18 US NHANES

	Asian (n = 760)	Black (n = 1,162)	Hispanic (n = 1,324)	White (n = 1,646)
	OR (95% CI)	OR (95% CI)	OR (95% CI)	OR (95% CI)
**Age, years**	1.03 (0.99, 1.06)	1.00 (0.99, 1.02)	1.01 (0.99, 1.03)	1.01 (1.00, 1.03)
**Marital status**				
Married	1.00 (ref)	1.00 (ref)	1.00 (ref)	1.00 (ref)
Never married, widowed, divorced, or separated	0.80 (0.50, 1.28)	0.97 (0.70, 1.35)	0.98 (0.67, 1.44)	1.08 (0.78, 1.49)
Living with a partner	0.97 (0.48, 1.93)	0.99 (0.59, 1.65)	1.00 (0.71, 1.41)	1.32 (0.83, 2.09)
**Education**				
≥College graduate	1.00 (ref)	1.00 (ref)	1.00 (ref)	1.00 (ref)
Some college or Associate in Arts degree	2.52 (1.76, 3.63)	2.00 (1.48, 2.70)	0.95 (0.66, 1.37)	2.39 (1.77, 3.24)
≤High school graduate/GED	2.43 (1.60, 3.68)	3.67 (2.51, 5.35)	1.55 (1.00, 2.41)	3.38 (2.32, 4.93)
**Employment**				
Employed	1.00 (ref)	1.00 (ref)	1.00 (ref)	1.00 (ref)
Unemployed	0.96 (0.64, 1.45)	0.72 (0.50, 1.06)	0.83 (0.64, 1.10)	1.03 (0.77, 1.39)
**Family income**				
High	1.00 (ref)	1.00 (ref)	1.00 (ref)	1.00 (ref)
Middle	1.06 (0.70, 1.62)	0.79 (0.57, 1.08)	1.49 (1.03, 2.14)	1.10 (0.84, 1.45)
Low	1.23 (0.64, 2.38)	1.10 (0.72, 1.70)	1.36 (0.90, 2.05)	1.31 (0.89, 1.93)
**Body mass index (BMI)**				
< 25 kg/m^2^	1.00 (ref)	1.00 (ref)	1.00 (ref)	1.00 (ref)
25 ≤ to < 30 kg/m^2^	0.79 (0.53, 1.18)	0.85 (0.59, 1.22)	0.77 (0.52, 1.13)	1.18 (0.84, 1.66)
≥ 30 kg/m^2^	0.87 (0.51, 1.51)	0.86 (0.64, 1.14)	1.39 (0.97, 2.00)	1.28 (0.98, 1.68)
**Children in the household**				
No children	1.00 (ref)	1.00 (ref)	1.00 (ref)	1.00 (ref)
Younger child(ren) only	2.08 (1.37, 3.17)	1.17 (0.82, 1.67)	0.99 (0.64, 1.54)	1.63 (1.11, 2.38)
Older child(ren) only	1.60 (1.02, 2.52)	0.90 (0.66, 1.22)	0.95 (0.64, 1.42)	1.32 (0.97, 1.79)
Younger and older children	2.61 (1.66, 4.08)	0.84 (0.58, 1.22)	1.30 (0.88, 1.92)	1.43 (0.96, 2.14)

CI, confidence interval; GED, general education development test; MVPA, moderate- and vigorous-intensity physical activity; OR, odds ratio

Exploratory analyses, which included the number of YC and the number of OC as predictor variables, showed a significant trend toward an increasing likelihood for physical inactivity with an increasing number of YC, but not with an increasing number of OC, among Asian women (Supplementary Table 1). White women with a higher number of YC also tended to have a higher likelihood of physical inactivity; however, the association was weaker than that among Asian women (Supplementary Table 1). When immigration status was considered, the multivariable logistic regression model showed that birthplace was not significantly associated with physical inactivity among Asian women (OR for those who were foreign-born vs. US-born = 0.89 [95% CI = 0.55, 1.44]).

In the multivariable linear regression analyses that included women (n = 2,759) who reported performing at least one minute of MVPA in a typical week, Asian women with YC engaged in 35 min/week less MVPA than their counterparts without children (p = 0.06). Among Black, Hispanic and White women, there were no significant differences in MVPA minutes between the YC and no-children groups (Table 4).

**Table 4:** Multivariable linear regression for weekly MVPA minutes (minutes/week) among woman participants aged 20–45 years in the 2011-18 US NHANES who engaged in at least one minute of MVPA in a typical week

	Asian (n = 474)	Black (n = 607)	Hispanic (n = 699)	White (n = 1015)
	Coefficient (95% CI)	Coefficient (95% CI)	Coefficient (95% CI)	Coefficient (95% CI)
Intercept	251 (154, 348)	240 (173, 308)	252 (159, 345)	242 (162, 322)
**Age, years**	0 (-3, 3)	-1 (-3, 1)	0 (-2, 3)	1 (-2, 3)
**Marital status**				
Married	Reference	Reference	Reference	Reference
Never married, widowed, divorced, or separated	21 (-15, 58)	-2 (-24, 21)	6 (-26, 39)	21 (-9, 51)
Living with a partner	63 (-3, 130)	-10 (-46, 25)	46 (17, 75)	-15 (-53, 22)
**Education**				
≥College graduate	Reference	Reference	Reference	Reference
Some college or Associate in Arts degree	-25 (-54, 4)	-23 (-52, 7)	-28 (-67, 10)	-17 (-45, 10)
≤High school graduate/GED	-19 (-51, 14)	-30 (-67, 7)	-18 (-56, 21)	-25 (-62, 11)
**Employment**				
Employed	Reference	Reference	Reference	Reference
Unemployed	41 (18, 64)	15 (-16, 47)	6 (-25, 37)	32 (7, 57)
**Family income**				
High	Reference	Reference	Reference	Reference
Middle	-23 (-52, 5)	-14 (-43, 16)	-26 (-60, 9)	1 (-21, 24)
Low	-20 (-69, 30)	-18 (-59, 22)	-25 (-65, 14)	21 (-16, 59)
**Body mass index (BMI)**				
< 25 kg/m^2^	Reference	Reference	Reference	Reference
25 ≤ to < 30 kg/m^2^	-5 (-40, 3)	-15 (-53, 23)	-25 (-58, 9)	-34 (-63, -5)
≥ 30 kg/m^2^	-47 (-73, -21)	-4 (-45, 36)	-24 (-58, 9)	-60 (-83, -36)
**Children in the household**				
No children	Reference	Reference	Reference	Reference
Younger child(ren) only	-35 (-72, 2)	7 (-30, 44)	-10 (-53, 33)	-22 (-22, 9)
Older child(ren) only	-14 (-51, 22)	0 (-33, 33)	4 (-34, 42)	-42 (-72, -11)
Younger and older children	-39 (-73, -4)	4 (-26, 34)	-21 (-52, 10)	-24 (-59, 11)

Weekly MVPA minutes were calculated as the sum of weekly transportation MVPA and weekly leisure-time MVPA.

CI, confidence interval; GED, general education development test; MVPA, moderate- and vigorous-intensity physical activity

## Discussion

This study found that approximately a third of American women aged 20–45 years living in a household without children (32%) did not participate in MVPA outside of work. Among American mothers of YC, the prevalence of physical inactivity outside of work was even higher at 43%, compared to American women without children. Our study also revealed racial and ethnic variations in the prevalence of physical inactivity outside of work: 24%, 28%, 42%, and 46% among Asian, White, Hispanic, and Black American women without children, respectively; and 43%, 41%, 43%, and 55% among Asian, White, Hispanic, and Black American women with YC only, respectively. Among women in the four racial and ethnic groups examined, the largest difference in MVPA minutes outside of work between women with YC only and without children was seen among Asian women. Specifically, for leisure-time MVPA, Asian mothers of YC on average engaged in 65 min/week, which was only about half of that performed by Asian women without children (117 min/week). We found no difference in MVPA engagement outside of work between Hispanic women with YC and without children.

PA levels have been shown to fluctuate throughout the life course [7]. Several studies [7, 8, 15] have demonstrated that the transition to motherhood is a significant life-course event that is associated with a PA reduction [8]. Within this literature, a study by Adamo et al. [15] is worth noting, as it is one of the few studies that used objective PA data from a large sample. Using a Canadian national sample, Adamo et al. [15] investigated the association between living with children of different ages in the same household and parents’ accelerometer-measured MVPA and found that mothers of YC engaged in fewer minutes of daily MVPA as compared to women living without children (18 vs. 23 min/day). The OR of physical inactivity was 3.2 (95% CI = 1.15, 9.09) for mothers of YC, compared to women living without children. While the study by Adamo et al. [15] provided evidence that women reduce PA in the transition to motherhood, it did not examine the heterogeneity of the association by racial and ethnic groups.

The present study is one of the first US national investigations to examine MVPA among mothers of YC by racial and ethnic groups. Consistent with the study by Adamo et al. [15], the present study found a higher likelihood of physical inactivity among American mothers of YC, as compared to American women without children. Further, this study demonstrated that among the four different racial and ethnic groups examined, Asian mothers of YC showed drastically lower MVPA, as compared to Asian women without children. This finding suggests that a PA reduction in the transition to motherhood may be particularly large among Asian American women. Data from the 2017–2020 Behavioral Risk Factor Surveillance System (BRFSS) showed that among American adults aged 18 years or older, Asian adults had the lowest prevalence of physical inactivity outside of work (20.1%), followed by White (23.0%), American Indian/Alaska Native (29.1%), Black (30.0%), and Hispanic (32.1%) adults [16]. However, when gender (women) and major life course events (motherhood) were considered, the present study revealed that many Asian mothers of YC (43%) were physically inactive; in fact, the prevalence of physical inactivity was similar between Asian and Hispanic mothers of YC. The present study also found that Asian mothers of OC had a similar prevalence of physical inactivity (41%) to Asian mothers of YC, suggesting that Asian women may not re-engage in transportation or leisure-time MVPA even after their child(ren) get older. This evidence points to a more nuanced understanding of PA among American women: while Asian women are relatively active when they do not have children, the transition to motherhood substantially increases their risk for physical inactivity, and this pattern may be sustained over time.

Although this study provided important evidence of a PA reduction during the transition to motherhood among Asian American women, the findings were limited by an inability to disaggregate Asian subgroups (e.g., Asian Indian, Filipino, or Chinese) due to the lack of information on Asian origin in the publicly available NHANES 2011-18 datasets. Using aggregated data could mask meaningful differences in a PA reduction during the transition to motherhood among women of Asian origin [17], especially considering reported leisure-time MVPA variations across Asian origin groups [18]. Limited data suggest that South Asian American women engage in extremely low levels of PA [19]. In a qualitative study by Dave et al. [20], South Asian American women reported that marriage and childbirth were key life events associated with their PA reduction. In future research, analyses by Asian origin group will be needed to better elucidate specific populations at highest risk for drastic PA reductions during the transition to motherhood.

Consistent with the existing literature [12, 16, 18, 21, 22], the present study found lower levels of MVPA engagement among Hispanic American women, compared to White American women. However, in contrast to our original hypothesis, we did not observe a significant association between the type of children living in the same household and MVPA among Hispanic women. Such a finding is somewhat inconsistent with the existing literature [23, 24]. A prior US-based study by Bautista et al. [23] reported that many Hispanic women with children reported “lack of childcare to be able to exercise” as a barrier to PA. Another US-based study by Marshall et al. [24] also reported that married or partnered Hispanic women were more likely to be inactive than married or partnered White women. While these findings support that Hispanic American women are more likely to be inactive in general, future research is warranted to better ascertain whether the transition to motherhood affects their PA behaviors.

Among the two PA domains assessed in this study, leisure-time PA and transportation PA, our findings suggest a potential substantial reduction in leisure-time PA during the transition to motherhood, particularly among Asian American women. Considering that mothers tend to spend a great amount of time, generally more time than fathers, on parenting and housework responsibilities [25–28], mothers often lack free time to spend engaging in PA [9–11]. A US-based study by Katz-Wise et al. [29] found that parents became more traditional in their gender-role attitudes and behaviors (gender-*differential* roles: husbands as breadwinners and wives as homemakers) during the transition to parenthood and such changes were greater among mothers than fathers. Therefore, allocating leisure time to PA among women could be a low priority in their transition to motherhood. And such changes could be greater particularly among Asian women, as traditional gender-role attitudes and strong cultural values for family life have been reported among Asian women [30]. For example, in a qualitative study by Dave et al. [20], South Asian American mothers reported marriage and parenting as barriers to PA, partly due to family disapproval and lack of awareness about the benefits of PA. Other studies [31, 32] have suggested that unfavorable attitudes toward PA (i.e., low value for PA) partly explains lower PA engagement among women from diverse racial and ethnic minority groups. Future research is warranted to better understand leisure-time PA changes in the transition to motherhood among Asian American women, which will inform culturally-tailored PA intervention programs for Asian American mothers.

Transportation PA, particularly walking, is a common and accessible means of achieving recommended PA levels [33] that can be incorporated into a woman’s daily routine. A study by Gao et al. [34] found that a childbirth life event was associated with an increase in transportation-related walking among Dutch adults. Scheiner [35] also reported an increase in transportation-related walking after a childbirth life event among German women; however, the increase was mostly to compensate for a decrease in transportation-related cycling. Somewhat contradictory to these prior findings from other countries, the present US study found that Black and Hispanic mothers of YC engaged in a similar level of transportation PA, compared to their counterparts living without children, while Asian and White mothers of YC engaged in even lower levels of transportation PA than their counterparts living without children. The variations across countries could partly be explained by differences in built environment characteristics (e.g., sidewalks) and parental leave policies [36, 37]. The present study further revealed that the difference in transportation MVPA minutes between women living with YC (22 min/week) and without children (45 min/week) was particularly large among White women. We suspect that there might be racial and ethnic differences or potential economic differences (e.g., vehicle ownership [34]) that impact the choice of transportation methods when traveling with YC. For example, families who own cars could frequently choose driving, rather than walking, to travel from place to place with YC. Given the mixed results reported in the literature, changes in transportation PA during the transition to motherhood should be further investigated in future prospective studies.

This study supports clinical practices for a routine evaluation of PA among first-time American mothers, particularly among Asian mothers, to identify those at risk for physical inactivity. Adopting the “critical windows theory,” researchers have suggested that the transition to motherhood can provide a window of opportunity to re-shape healthy PA habits [38, 39]. The findings of the present study suggest that, particularly among Asian American mothers, the new motherhood period may be a critical window to promote PA. Promoting PA among mothers of YC also could indirectly help promote PA among YC, through modeling PA behaviors and co-participation in PA [40, 41]. Therefore, PA interventions for mothers of YC have the potential to make great health impacts not only on mothers, but also on YC. Based on the theory of planned behavior, empirical data [42] showed that among new mothers, an affect-based behavioral belief (e.g., PA relieves stress) and a control belief (e.g., it is easy to do PA even if I am tired), were positively associated with participation in MVPA. Community-based physical activity programs are recommended for Asian American mothers, and the programs should be culturally tailored to integrate languages, specific cultural values, social norms and preferences, social supports, and gender roles. To design such tailored interventions, identifying target subgroup-specific resilience and risk factors for PA reduction during the transition to motherhood will be critical.

### Limitations

The limitations of this study include a cross-sectional design and aggregated analyses for Asian subgroups. This secondary analysis was unable to examine barriers to PA specific to mothers of YC because PA barrier data were not collected in the 2011-18 NHANES. Another limitation is that housework PA was not considered because the 2011-18 NHANES PAQ did not assess housework PA. Defining women living with YC in the same household as mothers of YC could have caused misclassification; for example, a woman living with a niece under age 5 years could have been misclassified as a mother of YC. This misclassification would have biased ORs away from the null hypothesis [43]. The study was also limited by the use of self-reported PA data, which are prone to measurement error. However, use of the PAQ data enabled us to assess domain-specific PA. Because response rates ranged from 52 to 71% in the 2011-18 NHANES cycles, selection bias might have affected the results, which is a threat to the internal validity of the study. Finally, unmeasured confounders could have affected the findings.

## Conclusions

In conclusion, this study found that American mothers of YC were less likely to engage in transportation or leisure-time MVPA than American women without children. This association was particularly stronger among Asian American women than Hispanic, Black, or White American women. The study results suggest that a PA reduction in the transition to motherhood may be particularly large among Asian American women, calling for targeted efforts for PA promotion among Asian American mothers of YC; e.g., culturally-tailored community-based physical activity programs for Asian American mothers.

### Electronic supplementary material

Below is the link to the electronic supplementary material.

**Supplementary Table 1**. Adjusted odds ratios of physical inactivity outside of work among woman participants aged 20-45 years in the 2011-18 US NHANES

## Data Availability

Data is publicly available at https://www.cdc.gov/nchs/nhanes/index.htm.

## Abbreviations

CI: Confidence interval
MVPA: Moderate- and vigorous-intensity physical activity
NHANES: National Health and Nutrition Examination Survey
PA: Physical activity
PAQ: Physical activity questionnaire
OC: Older children
OR: Odds ratio
YC: Young children
