# Supplementary material for: Racial and ethnic differences in physical activity among mothers of young children: 2011–2018 NHANES
Source: BMC Womens Health. 2023 Aug 18;23:439. doi: 10.1186/s12905-023-02591-x (PMC10439601; doi:10.1186/s12905-023-02591-x)
Supplement: Supplementary file 1 — Supplementary Table 1. Adjusted odds ratios of physical inactivity outside of work among woman participants aged 20-45 years in the 2011-18 US NHANES [file 12905_2023_2591_MOESM1_ESM.docx]

**Supplementary Data**

**Supplementary Table 1.** Adjusted odds ratios of physical inactivity outside of work among woman participants aged 20-45 years in the 2011-18 US NHANES

|  | **Asian**  **(n=760)** | **Black (n=1,162)** | **Hispanic (n=1,324)** | **White (n=1,646)** |
| --- | --- | --- | --- | --- |
|  | **OR (95% CI)** | **OR (95% CI)** | **OR (95% CI)** | **OR (95% CI)** |
| **The number of younger children living in the same household** | | | | |
| None | 1.00 (reference) | 1.00 (reference) | 1.00 (reference) | 1.00 (reference) |
| One | 1.64 (1.14, 2.34) | 1.02 (0.74, 1.42) | 1.19 (0.92, 1.53) | 1.23 (0.89, 1.71) |
| ≥Two | 2.36 (1.32, 4.19) | 1.13 (0.74, 1.72) | 1.27 (0.84, 1.93) | 1.59 (1.13, 2.25) |
| **The number of older children living in the same household** | | | | |
| None | 1.00 (reference) | 1.00 (reference) | 1.00 (reference) | 1.00 (reference) |
| One | 1.57 (1.03, 2.40) | 0.77 (0.58, 1.03) | 1.08 (0.80, 1.45) | 1.28 (0.91, 1.82) |
| ≥Two | 1.36 (0.90, 2.06) | 0.88 (0.65, 1.20) | 1.14 (0.84, 1.54) | 1.01 (0.70, 1.46) |

CI, confidence interval; OR, odds ratio.

OR was adjusted for age, marital status, education, employment, family income, and body mass index category.
